# The left atrial bacterial vegetative mass due to *Corynebacterium striatum* as a presentation of myxoma: a case report

**DOI:** 10.1186/s12879-017-2468-8

**Published:** 2017-05-26

**Authors:** Jun Xu, Qing Yang, Jun Li, Xia Zheng

**Affiliations:** 1 Intensive Care Unit, The First Affiliated Hospital, College of Medicine, Zhejiang University, 79 Qingchun Road, Hangzhou, 310003 People’s Republic of China; 2 Department of Clinical Laboratory, The First Affiliated Hospital, College of Medicine, Zhejiang University, Hangzhou, People’s Republic of China; 3 Department of Pathology, The First Affiliated Hospital, College of Medicine, Zhejiang University, Hangzhou, People’s Republic of China

**Keywords:** Infective endocarditis, *Corynebacterium Striatum*, Atrial myxoma, Bacterial vegetation, Case report

## Abstract

**Background:**

*Corynebacterium striatum i*s a member of the non-diphtherial corynebacteria, which are ubiquitous in nature and generally colonize the skin and mucous membranes of humans. Rarely, it causes infective endocarditis (IE). We report a case of rare left atrial bacterial vegetative mass due to *C. striatum* masquerading as a myxoma identified through a tortuous diagnostic process, and present a brief review of the relevant literature.

**Case presentation:**

We present a case of 63-year-old man who presented with progressively worsening dyspnea on exertion and lower leg edema, and was diagnosed with heart failure. Transesophageal echocardiography (TEE) revealed that the left atrium was filled with a 2.7 cm × 2.6 cm mass. The patient, who had no signs of infection or related risk factors, was suspected of having a left atrial myxoma clinically. After excising the mass, the histopathology suggested thrombus with no myxocytes. Postoperatively, a fever appeared and *C. striatum* was isolated from the blood cultures. Although antibiotics were used, the symptoms of heart failure worsened gradually and echocardiography revealed valve vegetation. The patient underwent a second operation because of IE. Surprisingly, the mass was confirmed to be a bacterial vegetation due to *C. striatum* based on Gram staining at a 1000× magnification, although this was not noted on routine pathological examination of the two surgical specimens.

**Conclusions:**

Physicians should be aware of *Corynebacterium* in blood cultures, which cannot simply be assumed to be a contaminant. A diagnosis of IE should be suspected, particularly in high-risk patients or those with an unexplained fever. Our patient had IE due to *C. striatum* with no risk factors. This case supports the diagnosis of IE using a combination of pathology and etiology.

**Electronic supplementary material:**

The online version of this article (doi:10.1186/s12879-017-2468-8) contains supplementary material, which is available to authorized users.

## Background

Infective endocarditis (IE) is a lethal disease that has undergone major changes in both host and pathogens over the past 20 years [1]. The clinical presentation includes a fever, new or changing heart murmur, embolic phenomena [2], heart failure, dyspnea, splinter hemorrhages, Roth spots, and glomerulonephritis. However, atypical clinical presentations of IE are common in the elderly or immunocompromised patients [3]. *Staphylococcus aureus* is the most common cause of IE in most of the industrialized world, whereas *Corynebacterium striatum* is a very rare cause.

Here, we present a patient with a left atrial bacterial vegetative mass due to *C. striatum* mimicking a myxoma, who was treated successfully with combined medical and surgical methods after a tortuous diagnostic process.

## Case presentation

**Fig. 1:**
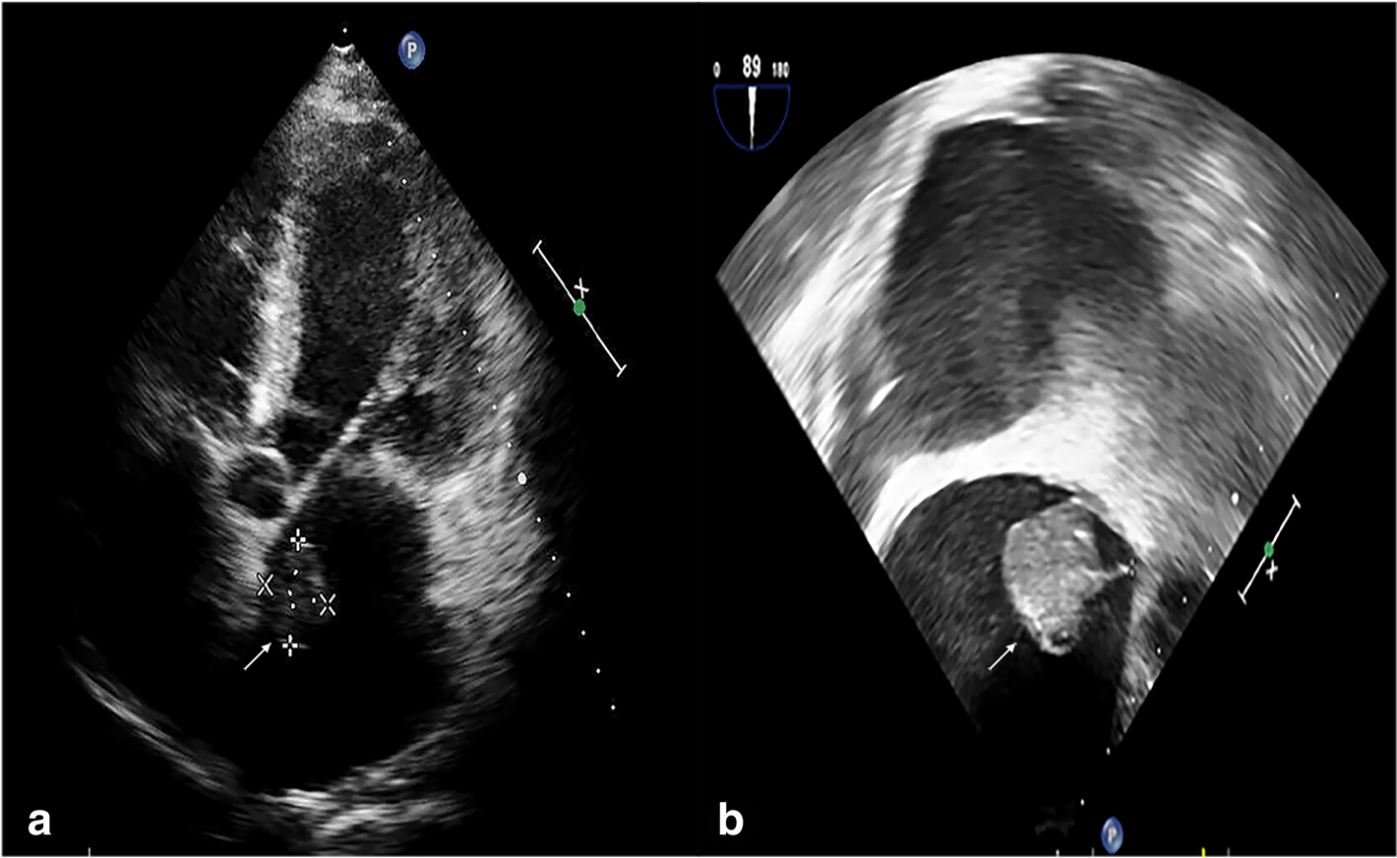
**a** Transthoracic echocardiography and (**b**)transesophageal echocardiography showing a 2.7 cm × 2.6 cm hypoechoic mass (arrow) attached by a thin stalk (measuring 0.15 cm in size) to the base of the atrial apex, freely swing within the left atrial cavity

**Fig. 2:**
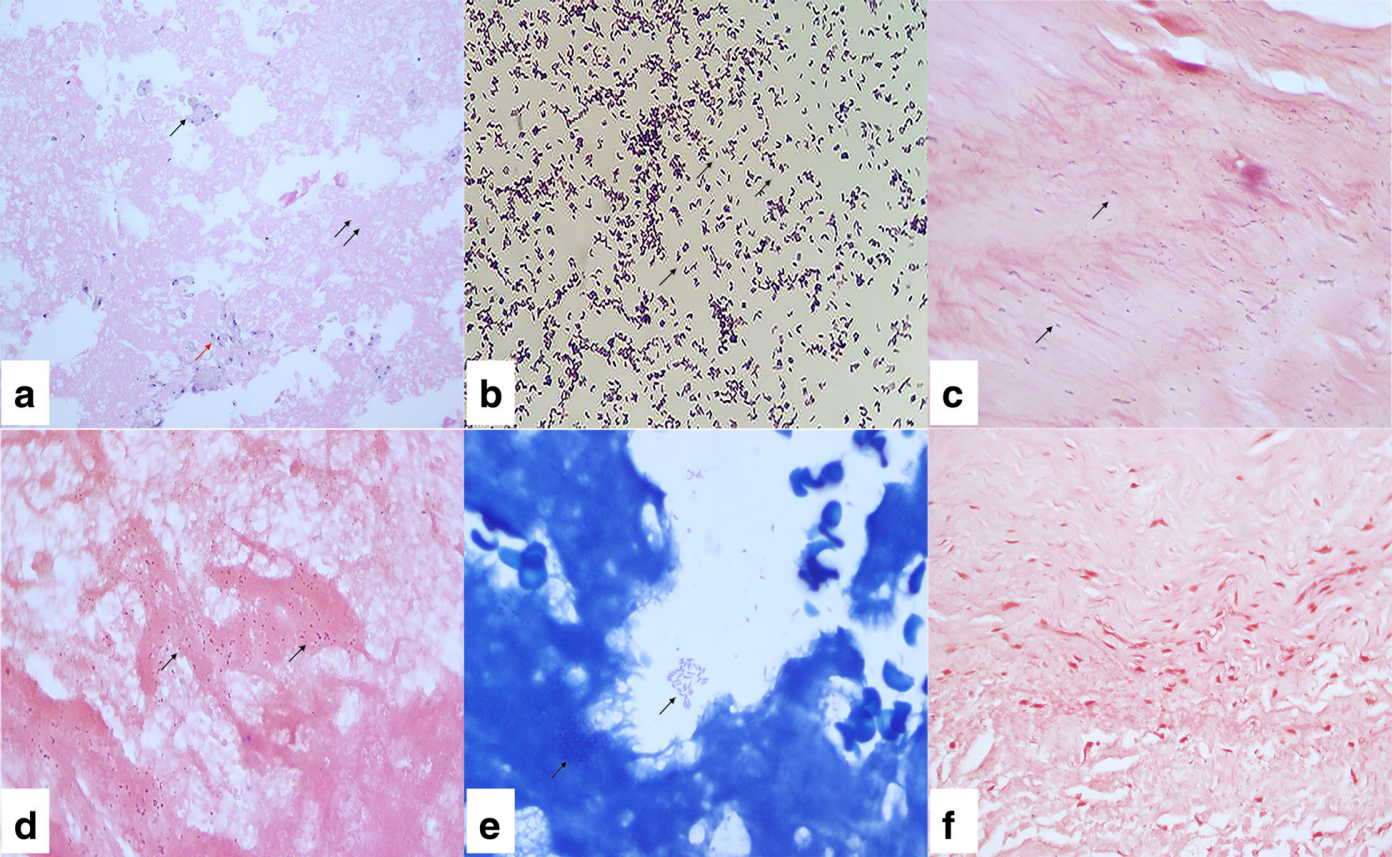
Pathological findings and staining of bacteria. **a** Hematoxylin and eosin (H&E) stain of the first excised mass showing large numbers of fibrinous exudate (double →) and chronic inflammation with a few mononuclear cells (→) and macrophage (→) (×200). **b** Gram’s staining of blood cultures showing large numbers of *C. striatum* (×1000). **c** Gram’s staining of the second excised vegetation showing *Corynebacterium* (×1000). **d** Gram’s staining and (**e**)Wright’s staining of the first excised mass for *Corynebacterium* (×1000). **f** *Corynebacterium* can not be found in second excised vegetation by Gram’s staining (×400)



A 63-year-old man with a history of hypertension and atrial fibrillation presented with progressively worsening dyspnea on exertion and aggravated lower leg edema for 1 month without a fever, coughing, or sore throat. He had no history of surgery. The symptoms of heart failure markedly improved after a 2-week treatment with diuretics in a local hospital. However, echocardiography showed a mobile mass in the left atrium; therefore, the patient was transferred to our hospital for further treatment. On admission, his vital signs were blood pressure of 145/90 mmHg, atrial fibrillation with a ventricular rate of 81 beats per min, and a body temperature of 36.3 °C. Laboratory tests showed a white blood cell (WBC) count of 3.8 × 10^3^/μL (reference range 4.0–10.0 × 10^3^/μL) with 44.7% neutrophils, and a serum hypersensitive C-reactive protein (hs-CRP) level of 8 mg/L (reference range 0.8–8 mg/L). His urine and feces examinations were normal. Transthoracic echocardiography (TTE) revealed a 2.7 cm × 2.6 cm left atrial mass that was suspected of being a myxoma (Fig. 1a). TEE also revealed a 2.7 cm left atrial mass attached by a thin stalk (0.15 cm) to the base of the atrial apex, swinging freely within the left atrial cavity (Fig. 1b and Additional file 1). The patient was managed surgically. The mass was confirmed to originate from the atrial apex and appeared grossly myxoid and fragile. Surprisingly, however, the histopathology of the specimen showed no characteristic stellate mesenchymal cells to support the diagnosis of myxoma. Instead, it revealed a fibrinous exudate and chronic inflammation with a few mononuclear cells and a macrophage reaction (Fig. 2a). Therefore, the mass was considered to be a left atrial thrombus.

**Additional file 1:** Video that demonstrates a 2.7 cm left atrial mass attached by a thin stalk to the base of the atrial apex, which was extremely similar to the myxoma, freely swing within the left atrial cavity, 7 s, 6.8 MB. (MP4 6644 kb)


Despite the preventive use of cefuroxime sodium (1500 mg every 12 h), his temperature increased to 39.5 °C on the fourth day. The WBC count increased to 9.5 × 10 [3]/μL with 85.9% neutrophils and his hs-CRP level rose to 183.9 mg/L. Four sets of blood cultures were drawn. Urgent computed tomography (CT) of the chest showed bilateral pulmonary infiltrates with sputum culture positive for *Acinetobacter baumannii*. Consequently, cefoperazone, sulbactam, and fosfomycin were started. Nevertheless, his fever recurred for 2 weeks. The blood cultures yielded *C. striatum* (Fig. 2b) that was resistant to ceftriaxone, penicillin, meropenem, and clindamycin and sensitive to vancomycin. Instead of vancomycin, linezolid was chosen to treat the *C. striatum* bacteremia after considering the elevated creatinine level of 117 μmol/L (reference range 59–104 μmol/L). However, the symptoms of heart failure worsened gradually and echocardiography revealed valve excrescence and severe aortic insufficiency. Therefore, the patient underwent surgical management and treatment with 500 mg daptomycin daily. A double valve replacement was performed. At surgery, a 1 cm × 1 cm vegetation was seen at the left coronary cusp of the aortic valve, with perforation of the right coronary cusp of the aortic valve and moderate mitral regurgitation. The fever subsided rapidly and six sets of blood cultures obtained postoperatively were negative. Histological examination of the new vegetation indicated large numbers of *Corynebacterium* on Gram staining (Fig. 2c) and vegetation cultures confirmed that the bacterium was *C. striatum*. We also found the same *Corynebacterium* on the first surgical specimen using Gram and Wright’s staining (Fig. 2e). We speculate that the left atrial mass was an occult bacterial embolus and large amounts of bacteria entered the blood stream and induced the valve damage after the first operation. Subsequently, the patient received a 4-week course of intravenous daptomycin and was switched to oral linezolid for 3 weeks. The patient’s general condition markedly improved and he was discharged from our hospital.

## Discussion

IE is a potentially lethal disease with a low incidence (1.7–7.9 cases/100,000 inhabitants). *C. striatum* is a rare cause of IE, causing about 0.33% of all cases of IE [4]. As a member of the corynebacteria, *C. striatum* is a Gram-positive, aerobic, non-sporulating bacillus that grows slowly in cultures and is distributed in the skin and mucous membranes of normal hosts and hospitalized patients. It is one of the more commonly isolated coryneform bacteria in the clinical microbiology laboratory and is usually considered a contaminant because of its low virulence. However, *C. striatum* can cause not only IE but also a variety of different infections such as pneumonia, empyema, peritonitis, arthritis, keratitis, intrauterine infections, wound infection, breast abscess, and osteomyelitis [5]. Risk factors for *Corynebacterium* endocarditis include pre-existing cardiac disease, a history of bacterial endocarditis, and the presence of prosthetic devices [6]. Rufael et al. [7] reported the first case of native valve endocarditis due to *C. striatum*, which required a combination of medical and surgical treatments [7]. Of the 24 cases of *C. striatum* endocarditis found in PubMed (Table 1) [6–30], most showed a predilection for heart valves. In our case, however, *C. striatum* colonized the left atrial apex masquerading as a left atrial myxoma instead of attaching to the heart valves. Looking back on our data, there were three main potential causes of misdiagnosis. First, the patient had no classic symptoms of IE on admission. Second, both TEE and the surgical findings supported the diagnosis of left atrial myxoma. Third, the initial histopathology was misleading.

**Table 1:** Summarizing previously reported cases of *C. striatum* endocarditis

Reference	Age	Sex	Associated illness	Valve	Intervention	Outcome
8	76	M	None	Aortic	Medical	Died
7	54	M	Hypertension	Aortic	Medical and surgical	Survived
9	73	M	Pacemaker	Tricuspid	Medical and surgical	Survived
10	24	M	Ventricular shunt	Pulmonary	Medical	Survived
11	68	M	Hypertension	Mitral	Medical	Survived
12	72	F	Prosthetic valve	Aortic	Medical	Died
13	62	F	Prosthetic valve	Aortic	Medical	Survived
14	50	M	Mycotic aneurysm	Aortic	Medical and surgical	Survived
15	61	F	Rheumatic fever	Mitral	Medical	Survived
15	72	F	Prosthetic valve	Mitral	Medical	Survived
16	46	F	Hemodialysis	Tricuspid	Medical	Survived
17	68	M	Prosthetic valve	Mitral	Medical	Survived
18	69	F	Endometrial cancer	Mitral	Medical and surgical	Survived
19	77	F	None	Mitral	Medical	Survived
6	62	M	Hypertension	Aortic	Medical and surgical	Survived
20	73	F	Hypertension, chronic kidney disease, and diabetes mellitus	Mitral	Medical	Survived
21	83	M	Metastatic prostate cancer	Mitral	Medical	Died
22	71	M	Diabetes mellitus	Mitral	Medical and surgical	Died
23	71	F	Pacemaker prosthetic valve	Mitral	Medical and surgical	Survived
24	62	M	Cardiomyopathy, diabetes mellitus and osteomyelitis	Aortic	Medical and surgical	Survived
25	69	F	ANCA+ vasculitis	Mitral	Medical and surgical	Died
26	51	M	Pacemaker	Not described	Medical and surgical	Survived
27	56	M	Diabetes mellitus, chronic kidney disease, and osteomyelitis	Mitral	Medical and surgical	Died
28	78	M	Chronic kidney disease, diabetes mellitus and pacemaker	Tricuspid and right ventricular wall	Medical and surgical	Survived
29	53	F	None	Quadricuspid aortic	Medical and surgical	Survived

The category “definite IE based on clinical criteria” involves with at least two major criteria, or one major criterion and three minor criteria, or five minor criteria. Major criteria include blood culture positive for IE, evidence of endocardial involvement, echocardiogram positive for IE, and new valvular regurgitation. According to the clinical, echocardiographic and biological findings, as well as the results of serologies, the patient did not meet the modified Duke’s criteria for diagnosis of endocarditis when he was transferred to our hospital. However, two of the major Duke criteria were met after the first operation―the positive blood culture and echocardiographic findings―enabling a definitive diagnosis [31]. Looking back, *Corynebacterium* was present in the first surgical specimen in our case. Therefore, the left atrial mass should have been considered an occult bacterial vegetation. The *C. striatum* was likely completely surrounded by fibrous tissue, so the patient had no signs of infection until bacteria were released by the first surgery. Ori Elkayam et al. [22] pointed out that IE is the most common manifestation of *C. striatum*, particularly in patients with nosocomial risk factors. But it is a potential pathogen even in normal hosts with no risk factors, such as our patient. Therefore, in addition to the routine pathological examination, special stains such as Gram staining or Wright’s staining should be performed if a few macrophages are seen. When a patient with suspected myxoma develops an unmanageable fever postoperatively, physicians should be alert to the progress of IE and evaluate the possibility with a combined pathological examination and blood cultures. The pathological examination of resected tissue or embolic fragments remains the gold standard for the diagnosis of IE. Nevertheless, we fell into a trap in this case because no pathogens were found in the first or second surgical specimens on examination with a medical microscope at a 400× magnification (Fig. 2f). We confirmed that *C. striatum* in positive blood cultures was responsible for the IE and that this diagnosis was supported by the results of Gram staining and Wright’s staining of tissue specimens viewed at a 1000× magnification with an oil immersion lens. Daptomycin is an effective drug for *C. striatum* and has been used in some patients with endocarditis caused by this organism [28]. Combined antibiotic treatment and surgery were performed because of the uncontrolled infection and severe aortic regurgitation based on the ESC guidelines [32]. The patient had a slow, uneventful recovery.

## Conclusions

*Corynebacterium* in positive blood cultures cannot simply be assumed to be a contaminant. The diagnosis of IE should be suspected, particularly in high-risk patients or those with an unexplained fever. Gram staining can provide additional support for a diagnosis of IE, particularly when the pathological examination implies an inflammatory response, and we should consider the size of the pathogenic bacteria and select the appropriate magnification when examining slides. This can guide postoperative antibiotic use and reduce the risk of a second surgery. In addition, it is necessary to make a suitable standard analysis protocol of cardiac masses to include thorough microbiological analysis, which can reduce misdiagnosis and improve the standard of care.

## Abbreviations

*C. striatum*: Corynebacterium striatum
CT: Computed tomography
hs-CRP: Hypersensitive C-reactive protein
IE: Infective endocarditis
TEE: Transesophageal echocardiography
TTE: Transthoracic echocardiography
WBC: White blood cell counts

## References

[1] Duval X, Delahaye F, Alla F (2012). Temporal trends in infective endocarditis in the context of prophylaxis guideline modifications: three successive population-based surveys. J Am Coll Cardiol.

[2] Thuny F, Di Salvo G, Disalvo G (2005). Risk of embolism and death in infective endocarditis: prognostic value of echocardiography: a prospective multicenter study. Circulation.

[3] Pérez de Isla L, Zamorano J, Lennie V, Vázquez J, Ribera JM, Macaya C (2007). Negative blood culture infective endocarditis in the elderly: long-term follow-up. Gerontology.

[4] Muñoz P, Kestler M, De Alarcon A (2015). Current epidemiology and outcome of infective Endocarditis: a multicenter, prospective, cohort study. Medicine (Baltimore).

[5] Severo CB, Guazzelli LS, Barra MB, Hochhegger B, Severo LC (2014). Multiple pulmonary nodules caused by Corynebacterium Striatum in an immunocompetent patient. Rev Inst Med Trop Sao Paulo.

[6] Belmares J, Detterline S, Pak JB, Parada JP (2007). Corynebacterium endocarditis species-specific risk factors and outcomes. BMC Infect Dis.

[7] Rufael DW, Cohn SE (1994). Native valve endocarditis due to Corynebacterium Striatum: case report and review. Clin Infect Dis.

[8] Markowitz SM, Coudron PE (1990). Native valve endocarditis caused by an organism resembling Corynebacterium Striatum. J Clin Microbiol.

[9] Melero-Bascones M, Muñoz P, Rodríguez-Créixems M, Bouza E (1996). Corynebacterium Striatum: an undescribed agent of pacemaker-related endocarditis. Clin Infect Dis.

[10] Tattevin P, Cremieux AC, Muller-Serieys C, Carbon C (1996). Native valve endocarditis due to Corynebacterium Striatum: first reported case of medical treatment alone. Clin Infect Dis.

[11] Juurlink DN, Borczyk A, Simor AE (1996). Native valve endocarditis due to Corynebacterium Striatum. Eur J Clin Microbiol Infect Dis.

[12] de Arriba JJ, Blanch JJ, Mateos F, Martínez-Alfaro E, Solera J (2002). Corynebacterium Striatum first reported case of prosthetic valve endocarditis. J Inf Secur.

[13] Houghton T, Kaye GC, Meigh RE (2002). An unusual case of infective endocarditis. Postgrad Med J.

[14] Kocazeybek B, Ozder A, Kucukoglu S, Kucukates E, Yuksel H, Olga R (2002). Report of a case with polymicrobial endocarditis related to multiresistant strains. Chemotherapy.

[15] Stoddart B, Sandoe JAT, Denton M (2005). Corynebacterium Striatum endocarditis masquerading as connective tissue disorders. Rheumatology (Oxford).

[16] Shah M, Murillo JL (2005). Successful treatment of Corynebacterium Striatum endocarditis with daptomycin plus rifampin. Ann Pharmacother.

[17] Mashavi M, Soifer E, Harpaz D, Beigel Y (2006). First report of prosthetic mitral valve endocarditis due to Corynebacterium Striatum: successful medical treatment. Case report and literature review. J Inf Secur.

[18] Tibrewala AV, Woods CJ, Pyrgos VJ, Ruiz ME (2006). Native valve endocarditis caused by *C. striatum*. Scand J Infect Dis.

[19] Elshibly S, Xu J, Millar BC, Armstrong C, Moore JE (2006). Molecular diagnosis of native mitral valve endocarditis due to Corynebacterium Striatum. Br J Biomed Sci.

[20] Marull J, Casares PA (2008). Nosocomial valve endocarditis due to corynebacterium striatum: a case report. Cases J.

[21] Bhat Y, Bal AM, Rochow S, Gould IM (2008). An unusual case of Corynebacterium Striatum endocarditis and a review of the literature. Int J Infect Dis.

[22] Boltin D, Katzir M, Bugoslavsky V (2009). Corynebacterium Striatum--a classic pathogen eluding diagnosis. Eur J Intern Med.

[23] Oliva A, Belvisi V, Iannetta M (2010). Pacemaker lead endocarditis due to multidrug-resistant Corynebacterium Striatum detected with sonication of the device. J Clin Microbiol.

[24] Batalla AS, de La Blanchardière A, Vergnaud M, Dargère S, Verdon R (2011). Recurrent Corynebacterium Striatum endocarditis, secondary to osteomyelitis. Med Mal Infect.

[25] Deligeoroglou E, Fotaki P, Kokkalis D, Creatsas G (2001). Description of 8 cases with gonadal dysgenesis syndrome type 46XY. Akush Ginekol (Sofiia).

[26] Knox KL, Holmes AH (2002). Nosocomial endocarditis caused by Corynebacterium amycolatum and other nondiphtheriae corynebacteria. Emerging Infect Dis.

[27] Tran TT, Jaijakul S, Lewis CT (2012). Native valve endocarditis caused by Corynebacterium Striatum with heterogeneous high-level daptomycin resistance: collateral damage from daptomycin therapy?. Antimicrob Agents Chemother.

[28] Fernández Guerrero ML, Molins A, Rey M, Romero J, Gadea I (2012). Multidrug-resistant Corynebacterium Striatum endocarditis successfully treated with daptomycin. Int J Antimicrob Agents.

[29] Fernández Guerrero ML, Robles I, Nogales MDC, Nuevo D (2013). Corynebacterium Striatum: an emerging nosocomial drug-resistant endocardial pathogen. J Heart Valve Dis.

[30] Mizoguchi H, Sakaki M, Inoue K (2014). Quadricuspid aortic valve complicated with infective endocarditis: report of a case. Surg Today.

[31] Li JS, Sexton DJ, Mick N (2000). Proposed modifications to the Duke criteria for the diagnosis of infective endocarditis. Clin Infect Dis.

[32] Habib G, Lancellotti P, Antunes MJ (2015). 2015 ESC guidelines for the Management of Infective Endocarditis: the task force for the management of infective Endocarditis of the European Society of Cardiology (ESC). Endorsed by: European Association for Cardio-Thoracic Surgery (EACTS), the European Association of Nuclear Medicine (EANM). Eur Heart J.

